# Interpersonal violence amongst primary health care patients in Lesotho: A qualitative study of the reasons for assault

**DOI:** 10.4102/phcfm.v5i1.473

**Published:** 2013-06-24

**Authors:** Kabilabe N.W. Ngobale, Gboyega A. Ogunbanjo, Olufemi B. Omole

**Affiliations:** 1 Department of Family Medicine and Primary Care, University of Limpopo, South Africa

## Abstract

**Background:**

Interpersonal violence is a common cause of morbidity and mortality. The incidence of and weapons used in interpersonal violence vary amongst countries and may even vary within regions of a country. Substance abuse, including alcohol, has been linked to interpersonal violence, but other socio-economic factors, cultural and traditional practices may also influence the perpetration of violence.

**Methods:**

In 2002, a qualitative study was conducted to explore the experiences of physically-assaulted victims of interpersonal violence at a local clinic in Leribe district of the Kingdom of Lesotho.

**Results:**

Aggravating factors for interpersonal violence included jealousy, unemployment, availability of weapons, substance abuse and poor levels of education. Interpersonal violence was ameliorated by family interventions, reporting to the authorities, seeking protection from assailants and religious assistance. Most interpersonal violence occurred during the night and on weekends. The head and neck regions are the most common anatomical sites of injury.

**Conclusion:**

Emotional and socio-cultural factors aggravate interpersonal violence in Lesotho, whilst family and religious interventions ameliorate it. Legislation addressing the protection of victims needs to be enacted, and community agencies dealing with interpersonal violence should be established. Studies which assess the requirements and the feasibility of intervention programs are also needed in the kingdom of Lesotho.

## Abstrait

**Les patients victimes de violence interpersonnelle dans les centres de soins de santé primaires au Lesotho: Étude qualitative des motifs d'agression**

**Présentation:**

La violence interpersonnelle est une cause de morbidité et de mortalité courante. L'incidence de la violence interpersonnelle et les armes utilisées dans ces actes varient d'un pays à l'autre, et peuvent même varier entre des régions d'un même pays. L'abus de substances, notamment d'alcool, a été lié à la violence interpersonnelle, mais d'autres facteurs socioéconomiques, culturels et associés aux pratiques traditionnelles peuvent également influencer la perpétration d'actes de violence.

**Méthodes:**

En 2002, une étude qualitative a été réalisée afin d’étudier les expériences des victimes de violence interpersonnelle physiquement agressées dans un centre médical local du district de Leribe, dans le Royaume du Lesotho.

**Résultats:**

Les facteurs aggravants de la violence interpersonnelle sont la jalousie, le chômage, la disponibilité d'armes, l'abus de substances et les faibles niveaux d’éducation. La violence interpersonnelle était améliorée par l'intervention des familles, leur dénonciation aux autorités, le recours à une protection contre les agresseurs et l'assistance religieuse. La majeure partie des actes de violence interpersonnelle se produisait la nuit et le week-end. Les régions de la tête et du cou sont les parties du corps les plus souvent affectées.

**Conclusion:**

Au Lesotho, des facteurs d'ordre émotionnel et socioculturel aggravent la violence interpersonnelle, et les interventions des familles et religieuses contribuent à y remédier. Une loi relative à la protection des victimes doit être promulguée, et des organisations communautaires traitant la violence interpersonnelle devraient être créées. Des études évaluant les exigences et la faisabilité des programmes d'information sont également nécessaires au Royaume du Lesotho.

## Introduction

Violence is one of the major causes of morbidity and mortality in the world,^1^ and is defined as:

[*T*]he intentional use of physical force or power (threatened or actual) against oneself, another person or against a group or community, that either results in or has a high likelihood of resulting in injury, death, psychological harm, mal-development or deprivation.^2^

In 2000, 1.6 million people died from violence-related causes world-wide.^3^ Violence is also common in Lesotho and is associated with significant morbidity and mortality.^4^ Young people between the ages of 20 and 49 years are most vulnerable and most physical assaults are inflicted by the use of traditional sticks, which forms part of the Basotho traditional costume.^4^ In addition, sexual violence against women is rife in Lesotho, with about 25% of women having being forced to have sex by intimate partners.^5^

Interpersonal violence (IPV) is defined as violence that occurs between people who know each other and is a subtype of violence. IPV is subdivided into family, intimate partner and community violence.^6^ It has been referred to as an accepted ‘societal norm’ in that some societies believe that some individuals have the right to violate others.^7^ A South African study found that a third of female respondents acknowledged that when a man beats his partner, it was an expression of love. Men are thus traditionally regarded as having the right to inflict physical punishment on their wives. In the same context, violence is sometimes considered as being a ‘tolerable way’ of solving differences. Little or no effort is therefore invested into preventing or intervening in IPV, leading to serious consequences such as deaths, injuries, destabilisation of family units, heavy workload on health workers, and added costs to health care and social welfare.^7^

Though there are many factors which influence the perpetuation of physical violence,^8, 9^ alcohol and drug use, including tobacco use, remain important factors in the overall causation of violence world-wide.^10, 11^ Regarding sexual abuse, factors such as being in a marriage relationship and having formal education are protective, and close proximity to areas of campaign against sexual abuse has been reported to increase the likelihood of victims reporting the incident.

Lesotho is a small mountainous country which is completely landlocked by the Republic of South Africa. It has a population of just over 2 million people. The population distribution of the country is 25% urban and 75% rural. Sesotho is the main language spoken by most people and is also the first official and administrative language. The country is divided into 10 administrative districts, of which Leribe district is one. Maputsoe Filter Clinic in the Leribe district is a primary care clinic situated 20km from the Motebang Government Hospital (the referral hospital). It shares a border-post with Ficksburg in the eastern part of the Free State, South Africa, and is the second-largest industrial town in Lesotho after Maseru, the political capital.

About 200 patients are attended to daily at Maputsoe Filter Clinic, with physical injuries arising from IPV being seen frequently, especially at nights and over the weekends. At the time of the study, there were no current data on IPV available in Lesotho. Despite the significant morbidity and mortality that may accompany IPV, very few studies have been conducted in Lesotho to provide a clear understanding of IPV in this setting. Of the few studies that were available electronically, one investigated the patterns of injuries, two others focused on the sexual aspects of IPV and one on school learners’ experience of violence. In order to gain a deep understanding of IPV and to inform the development of context-appropriate interventions in Lesotho, it was imperative to identify the issues associated with IPV. This study therefore explored the understanding of physically-assaulted victims who presented to Maputsoe Filter Clinic regarding the reasons for their being assaulted. It is hoped that, since no previous study had been conducted on IPV in this health care setting, the findings of this study will be critical in developing appropriate interventions at the primary health care (PHC) level.

## Research method and design

A qualitative study using a one-on-one interview technique was conducted. In order to explore the reasons for being assaulted, as reported by victims of IPV, it was deemed appropriate to use a qualitative design. Qualitative methods are appropriate for exploring people's perceptions, views and behaviours which are grounded on their lived experiences.^12^ The goal of qualitative research is to develop concepts which help people to understand social phenomena in their natural settings.^13^

Prior to the commencement of the study, a pilot study was conducted in order to test the trained nurse's interviewing capacity and to evaluate the interview process. The results of the pilot study were not included in the study.

### Procedure

Participants who were physically assaulted and assessed by the researcher and the nurse interviewer as information-rich patients were recruited to participate in the study between May and July 2002. The recruitment was performed only when the PHC nurse attached to the clinic was on duty in order to prevent interference with service delivery, as the researcher was the only doctor in the clinic's service. Recruitment of the physically-assaulted victims of IPV continued until data saturation was reached. By this point, 10 participants had already been interviewed. Eligible physically-assaulted victims of IPV who were 18 years and older, assaulted within the previous four weeks, spoke Sesotho fluently, and who gave written consent were included in the study. The age of 18 years was chosen so as to allow participants to consent without the assistance of a legal guardian or parent.

Prior to all interviews, participants were assessed to be clinically stable and all appropriate care was instituted. An exploratory question was posed to each participant. The exploratory question was: ‘Hobaneng ha o nahana hore o ile oa otloa oa ba ntsoa likosi?’ Translated into English, this means: ‘Why do you think you were physically assaulted?’ The interviews were then facilitated by the nurse-interviewer who was trained on qualitative research interviewing techniques. The interview was facilitated using open-ended questions, silence, clarification, reflection and summaries. Field notes were also kept for non-verbal cues by the researcher. Each interview was audiotaped and later transcribed verbatim in Sesotho by the nurse-interviewer, followed by translation of the text into English by a lecturer in the Language department of the National University of Lesotho. Emerging themes were organised into categories using the ‘cut and paste’ process. A combined list of themes representing the participants’ perceptions of the reasons for their being physically assaulted was developed and compiled by the researcher. The field notes were used to support the process of thematic analysis. Triangulation of findings was performed through independent analysis by the first researcher, compared with that of the academic supervisor and a third colleague, who also served as a peer reviewer. Member checks were performed by feedback of the findings to the participants for verification of the transcribed data and identified themes.

## Ethical considerations

Ethical clearance (MP106/2001) and permission to conduct the study were obtained respectively from the Research Ethics and Publication Committee of the Medical University of Southern Africa (now University of Limpopo – Medunsa Campus) and the District Medical Officer of Leribe district.

## Results

### Participants’ characteristics

Ten patients were purposively selected as information-rich participants: seven females and three males. Other characteristics of the participants are shown in Table 1.

**TABLE 1:** Demographic data of the participants.

Participants	Age (Years)	Sex	Marital status	Occupation	Education level	Religion
P1	44	Male	Married	Self-employed	Grade 4	Jehovah's Witness
P2	42	Female	Married	Server at restaurant	Grade 9	Roman Catholic Church
P3	23	Female	Married	Server at shop	Grade 9	Anglican Church
P4	52	Male	Married	Farmer	Grade 2	Lesotho Evangelical Church
P5	29	Female	Married	Machinist	Grade 7	Roman Catholic Church
P6	23	Female	Divorced	Sales woman	Grade 7	Lesotho Evangelical Church
P7	36	Female	Married	Self-employed	Grade 10	Roman Catholic Church
P8	33	Female	Divorced	Housewife	Grade 5	Roman Catholic Church
P9	36	Male	Married	Self-employed	Grade 7	Roman Catholic Church
P10	28	Female	Widow	Sales woman	Grade 9	Apostolic Faith Mission

*Source:* Data gathered during interviews

The main findings of the study were clustered into two major categories – issues which contributed to the incidents of IPV and those which ameliorated the IPV. The themes in each category represent the main findings of this study and are summarised in Table 2.

**TABLE 2:** Themes and sub-themes.

Themes	Sub-themes
Contributory factors	Jealousy Robbery Unemployment and low level of education Availability of weapons Patriarchal behaviour and peer pressure Not reported to authorities Lack of legislation on domestic violence and police's inefficiency Previous assaults Influences of substance abuse
Ameliorating factors	Reporting to the authority Family intervention Fear of living with their assailants Religious assistance
Time of the violence	Weekend and evening
Anatomical site	Head and neck

*Source:* Data gathered during interviews

### Contributory factors

#### Jealousy

The assailants’ feelings of jealousy were a major source of violence in seven of the 10 participants interviewed. Jealousy predominated more in incidents where the victim and assailant were in intimate relationships. Though living apart, divorced and/or separated couples also expressed jealousy when they met a former partner with a new lover, as demonstrated by following quote:

‘He came to my place and found me with another man in my house. He once said that my love for him is not like in the past. Now, he has found this man in my house’. (P10, Female, 28)

Another participant who was separated from her husband initiated the confrontation because she believed her husband was having extramarital affairs and said:

‘I heard that these girls are brought there for my husband to choose [*from*]’. (P8, Female, 23)

Yet another participant felt her husband thought she was cheating on him and assaulted her:

‘He said I have gone there to sleep around with those men because I talked to those men in the tavern in his presence. He said where is that boyfriend of yours [*to whom*] you were talking?’ (P6, Female, 23)

Others reported that they got assaulted because their assailant was envious of their employment status, as their partners were not employed:

‘He is jealous that I am working and he is not’. (P3, Female, 23)

Jealousy was also encountered amongst business partners. One participant stated:

‘His customers have left him to buy at my shebeen so he is jealous. He once told me that he wants to talk to my husband so that they can be shareholders [*partners*]’. (P7, Female, 36)

#### Robbery

Two circumstances of violence were related to robbery:

‘He put his hand in his jacket and took out a gun. They hit me with me with those guns all over my body … They searched in my pockets. They took my wallet, cell-phone and some other keys’. (P1, Male, 44)

The second robbery victim (P9) reported that being armed with weapons made the assailants feel powerful, but she was not afraid of them.

#### Unemployment and low level of education

The participants reported that most of their assailants were unemployed (only two assailants were employed), and reckoned that the financial dependence of the assailant on the victims predisposed them to being assaulted:

‘Since the time we came together he was not working. I was the only one working’. (P6, Female, 23)

All but one victim were employed, but earned an average income of only R140 per week, probably because of their low educational attainment:

‘He is not working, he ended in standard five’. (P4, Male, 52)

Victims as well as assailants involved in IPV in this study had, on average, reached grade five. None of the interviewees had completed secondary education. Regarding the assailants, one had a Masters degree in one of the social sciences, whilst the majority had only reached grade four.

#### Availability of weapons

This contributed to the assailants’ assaulting their victims. Weapons used included guns, knives, sticks, stones, bottles, iron rod, other available domestic objects and human fists. Except for the incidents involving robberies, where guns were used, most assailants used weapons which were freely available to them at the time of assault. These weapons were used singly or in combination, as reported by a participant who said:

‘He beat [*me*] on the face with his fist, he hit me with his foot on the abdomen, he took that Prime stove [*in the kitchen*] and threw it on me. I found him also holding a big stick [*and*] he even had a knife’. (P3, Female, 23)

Guns were used in two incidents, whilst belt, stones, iron rod and bottles were used in eight cases. One participant said:

‘He put his hand inside the pocket and pulled out a gun …’ (P6, Female, 23)

Sticks, as part of the Basotho traditional costume, were used by the assailants in five cases:

‘He hit me with a stick’. (P2, Female, 42)

#### Patriarchal behaviour and peer pressure

Participants felt that men, as family leaders, took advantage of women in order to demonstrate their masculinity, and showed no accountability to anyone:

‘He fears no one’. (P3, Female, 23)

Another participant reiterated this lack of accountability, saying:

‘I have seen that these days he can do anything [*he likes*] but he will never ask for forgiveness …’ (P5, Female, 29)

Others reported that their assailants acted out of peer pressure from friends:

‘I have heard that there are some of his friends that are encouraging him to be disrespectful …’ (P4, Male, 52)

On the other hand, some participants thought that their assailants perceived them (the victims) as encouraging their family members to disrespect them (the assailants), thus leading to their being assaulted:

‘[*He thinks*] I am the one who is teaching his wife [*and his son*] all these bad behaviours’. (P2, Female, 42)

#### Lack of reporting

Three out of the 10 incidents in this study were not reported to the authorities (either the police or the local traditional chief) and alternative means of dealing with the violence were explored. The participants who did not report the incidents preferred their families’ interventions:

‘I will not go to the police before I go to his brother to get in between and solve our problem, like he tried to help us previously’. (P8, Female, 33)

Another stated:

‘What I think is to go home and tell his parents and my own [*parents*] and ask them to come in between. But if they do not help me, I will see if I can take other measures’. (P5, Female, 29)

Additionally, the fear of harming the assailant was also considered by participants, one of whom said:

‘I am afraid to see him arrested’. (P8, Female, 33)

Nevertheless, the same interviewee added that if this approach failed, she would have no choice but to report the assault to the police.

#### Lack of legislation on domestic violence and police's inefficiency

These factors were seen to encourage the perpetuation of IPV and were a source of frustration for the victims. Most victims knew that they should report incidents of IPV to the police:

‘I want him to be arrested because my life is in danger. It is my wish to see him being arrested’. (P6, Female, 26)

However, there was no official legislative framework to guide officials in their dealings with IPV, especially with regard to the availability of places of safety for immediate protection of the victims from their assailants. One participant reported that she had to stay in the abusive relationship because:

‘[*T*]here is no place I can stay [*in*]’. (P5, Female, 29)

Participants often run to relatives for safety:

‘I managed to escape to his brother's house’. (P3, Female, 23)

Most victims reported their assailants to the police but some were sceptical of the effectiveness of the police when dealing with the incidents. There was a widespread perception that the assailants believed that the police would not do anything:

‘He usually says that if he is arrested, he will not even sleep [*in jail*] for a day’. (P10, Female, 28)

Another participant said:

‘He said I can go to the police officers, he does not fear them’. (P6, Female, 23)

Despite this, she reported the assailant to the police. However, another participant was discouraged from going to the police to lay a complaint. In the end, she decided to resolve the matter amicably with the help of her parents:

‘Since I have told my parents all this, I wanted [*it*] to be solved by them’. (P10, Female, 28)

#### Previous assaults

Most of the participants (seven out of 10) reported that the current episode of assault was not the only one and that their assailants had assaulted them in the past:

‘It is the second time’*. (*P7, Female, 36)

Another reiterated:

‘This is not the first time. This [*violence*] started since last year when we had that conflict …’ (P5, Female, 29)

Three participants viewed the episodes of violence as being a normal part of life:

‘We have never lived peacefully with him since we are married’. (P3, Female, 23)

#### The influence of substance abuse

This factor was controversial and unclear. Two participants reported that substance abuse played a part in causing the violence:

‘All the time he is drunk. He could not [*have*] assaulted me if he was not drunk. He even uses dagga’. (P2, Female, 42)

However, the majority (seven of the 10 participants) believed that with or without substance use, if the assailant intended to harm them, he or she would do so:

‘No, he is not using any kind of drugs’. (P5, Female, 29)

Another reiterated that although ‘he [*the assailant*] is using alcohol a lot and even dagga’, the substance abuse had nothing to do with the act of violence:

‘He was not under the influence of any drugs. He did this purposely. The time he threw stones at me, he was sober’. (P4, Male, 52)

### Ameliorating factors

#### Reporting to authority

This was considered to be a means of seeking protection and allowing the law to be applied accordingly. The majority (seven participants) chose to report their assault to the police. These participants reported that their lives were in danger and that they wished to see their assailants arrested:

‘I told them that I am taking him to the police, nothing else’. (P2, Female, 42)

Another stated:

‘I think the prison will help him. I want him to be arrested’. (P4, Male, 52)

Two participants who were involved in robberies reported to the police with the aim of recovering what was stolen from them during the violence.

#### Family intervention

Three participants opted for family intervention as a means of settling the conflicts associated with IPV, rather than reporting the incidents to the police:

‘Since I have told my parents all this, I wanted [*it*] to be solved by them’. (P10, Female, 28)

Others were aware of the possibility of reporting the assault to the police but would rather try family intervention first. This is reflected in the statement of a participant who said:

‘What I think is to go home and tell his parents and my own [*parents*] and ask them to come in between. But if they do not help me, I will see if I can take other measures’. (P5, Female, 29)

A participant who was assaulted by his son indicated that he had tried family intervention in the past as part of his efforts to try and help his assailant:

‘I tried to call members of the family to try to talk to him but they failed. I even tried to call the elders of the family to try to talk to him …’ (P4, Male, 52)

#### Religious assistance

This was viewed by participants as being a strong and alternative support system when dealing with IPV. Despite having reported the assault by his son to the police, one participant still invited his church members to pray for his son (the assailant) with the hope that he would be rehabilitated and lead a good life:

‘Maybe spiritual healing could help, I even called members of my church to come and make a prayer for him’. (P4, Male, 52)

#### Fear of living with the assailants

The reported assault and violence were typified by intimidation from the assailants. This made some participants develop a fear of living in the same dwelling as their assailants and prompted them to seek protection elsewhere. Six out of the 10 interviewees shared this opinion:

‘[*N*]ow he has shown me that he can kill me. He has shown that he can kill me anytime … I think the prison will help him. I want him to be arrested’. (P4, Male, 52)

Another participant stated that:

‘[*t*]here is no guarantee that I will be safe there at my place’. (P5, Female, 29)

So, the victim went to report the assault to the police.

### The day, place and time of the violence

Most participants were assaulted within the vicinity of their homes during the weekends (from Friday to Sunday) and in the evenings:

‘I came back late from work on Saturday. I was outside there till 10:00PM, when he [*the assailant*] came and unlocked the house’. (P3, Female, 23)

Another participant reiterated:

‘It [*the assault*] was around 8:30PM when I intended to leave their place’. (P7, Female, 36)

However, some assaults took place during weekdays, outside of the homes and when the participants were alone, especially those involving robberies:

‘It was on Monday. When I left their place [*and*] I gently [*sic*] went out alone’. (P1, Male, 44)

### Anatomical sites

The head and neck was the region of the body most targeted by assailants and had more injuries than other parts of the body:

‘Injuries are on the head and below the right eye’. (P9, Male, 36)

Nine of the 10 participants reported the head as being the most common site of injury although other parts of the body were also involved, often at the same time:

‘[*The injuries are*] on the face. He was using his hand to beat me on the forehead, he used his gun to beat me and on the thighs he used his feet to kick me’. (P6, Female, 23)

## Discussion

The use of qualitative methodology in this study has enabled the exploration of the lived experience of patients at Maputsoe Filter Clinic Lesotho, so as to gain insight into the reasons for being physically assaulted. The findings suggest that various issues give rise to IPV and explain the reasons why the patients seen at this clinic were physically assaulted. These are clustered into themes and presented in Table 2.

Feelings of jealousy were reported as being a prominent contributory factor for the development of IPV in this study. The same factor has been reported as being an aggravating factor by Motlomelo and Sebatane, in a previous study conducted in three different districts of Lesotho.^14^ Most acts of jealousy were reported in intimate relationships when one partner perceives his or her interest, or position, to be threatened by the victim. However, it was even reported in some of the *non*-intimate relationships in the current study, as mentioned by the participant who was assaulted because the assailant reportedly lost out in a business competition.

Different weapons were used to inflict injuries on the victims in this study and their availability seemed to influence the assailants to commit violent acts. Most acts of violence involved the use of easily-available weapons in the home. However, those incidents involving robberies were associated with the use of more deadly weapons such as guns. The availability of weapons has also been highlighted as a contributory factor toward high levels of violence in other countries. Considering this link, more attention needs to be focused on defining what constitutes a weapon in Lesotho, given the free availability of traditional sticks (called ‘molamo’ in seSotho) and their importance as part of the traditional costume in Lesotho.^15^ This is crucial, as roughly half of the participants in this study reported the use of these sticks during the assaults. Similar findings were reported in a survey conducted at Quthing, Lesotho, where 55% of injuries were inflicted using traditional sticks.^4^ Sticks constitute part of the paraphernalia of the Basotho traditional dress but, in that context, they are not considered to be a weapon. Awareness and educational campaigns need to be rolled out in collaboration with traditional leaders in order to address the influence of tradition and culture on the perpetuation of interpersonal violence.

Most assailants in this study were unemployed and this has been identified, in a previous report by Cooper et al.^16^, as being a contributory factor for violence. Another study also found that domestic violence was predominantly found amongst illiterate women and that education might help in the prevention of domestic violence.^10^ The high rate of unemployment in this study may be a reflection of the low educational attainment of the assailants, which makes them less skilled and therefore less employable. Given that most assailants were men, being unemployed becomes a cultural challenge to them, as they are expected to be the providers (or breadwinners) within the traditional African setting. Their failure to fulfill this role makes them economically dependent on their female partners and may serve as a trigger for confrontations and violence. Interventions aimed at preventing IPV in Lesotho therefore need to consider innovative ways of addressing this high unemployment level amongst men.

Two participants in this study perceived robbery as being the motive for their attacks. This is consistent with the findings of a previous study in a similar setting in South Africa which showed that 23.7% of victims perceived the motive for their attack to be robbery.^17^ Most of the violence reported in this study occurred over the weekends. Weekends appear to be targeted most often for committing acts of violence, as has previously been described in the literature.^18, 19, 20^ Victims might be more vulnerable to being robbed over the weekend, after people have received their wages, and under the cover of the night, when opportunities for escape without being noticed are higher for the assailants. Incidents of violence often occur at night, as has been confirmed by previous reports.^17, 21^

At the time of the study, there was no official policy on IPV in Lesotho, a situation which hindered the effective management of victims and prosecution of assailants. There were also no referral routes to appropriate agencies which deal with IPV, including places of safety. The lack of easy access to law enforcement agencies and social support agencies placed the victims in a revolving cycle of assault. All victims of ongoing violence should have a safety plan and the safety of the victims must be a priority in the global management of IPV.^10^ Reporting incidents of violence to the authorities was another important management option for many victims but the lack of telephone hotlines in Lesotho is a serious limitation. In the United States of America, a nationwide 24-hour toll-free domestic violence hotline is available to help victims of domestic violence.^22^ This telephone service provides easy access for victims to report injury-related violence, helps to initiate the process of providing a place of safety for victims where needed and also helps to initiate the prosecution of assailants, as appropriate.

Religious beliefs may play a role in the way victims in this study addressed IPV. The expectation that prayer as an intervention can change the life of an assailant was expressed by one of the participants who stated:

‘I even called members of my church to come and make a prayer for him’. (P4, Male, 52)

This expectation has been expressed by Saunders, who commented that persons committed to Christ are expected to behave worthily and peacefully – implying that commitment to Christianity can change bad behaviours to acceptable ones.^23^

Although most assailants had been drinking alcohol prior to the assaults, most participants (victims) did not believe that substance use was a reason for the violence. Seven out of the 10 participants believed that the assailants had premeditated the incident and would have carried out the acts of violence even without alcohol and drug use. In Buenos Aires, Argentina, the pattern of deliberate violence did not seem to be associated with the influence of alcohol.^24^ However, although only a few participants in this study perceived substance abuse as being a reason for violence, alcohol abuse has been found to be associated with domestic violence in a number of previous studies.^25, 26, 27^ In reconciling the two stances, substance abuse may be a risk factor, but should not be a catch-all explanation for why violent acts are committed.

The head was the most common anatomical location of injuries. Similar findings were reported by Schreyer et al. where 43% of participants with assault-related injuries had injuries sustained to the head and the neck.^20^ Most IPV-related injuries displayed evidence of intent to kill or do serious bodily harm and are therefore found on the head, neck and trunk. Injuries to the head should thus be considered to be non-accidental, as unintentional injuries tend to be found in more peripheral regions of the body.^18^

## Limitations of the study

The findings reported in this study are based on self-reporting and are therefore prone to ‘information bias’. The qualitative design makes these findings unique to the context of the research and the findings are not able to be generalised. The purposive sampling and the recruitment of patients only when the nurse-interviewer was on duty predisposed the researcher toward missing other potentially well-informed patients who attended the clinic in her absence. Notwithstanding these limitations, this study provides an understanding of the reasons for assault amongst physically-assaulted patients in a typical PHC setting in Lesotho.

## Conclusion

A wide range of psychosocial, cultural, economic and policy issues contributed to the perpetuation of IPV amongst these patients, whilst reporting the incidents of IPV to the authorities and family, as well as religious interventions, were perceived to ameliorate IPV. Although substance use in the form of alcohol was common amongst assailants, the participants did not perceive their use as being a reason for IPV. The implementation of comprehensive policy guidelines and treatment protocols that take cognisance of the different risk factors is crucial to addressing IPV in Lesotho.
